# Research Screener: a machine learning tool to semi-automate abstract screening for systematic reviews

**DOI:** 10.1186/s13643-021-01635-3

**Published:** 2021-04-01

**Authors:** Kevin E. K. Chai, Robin L. J. Lines, Daniel F. Gucciardi, Leo Ng

**Affiliations:** 1 Curtin Institute for Computation, Curtin University, Perth, Australia; 2 School of Population Health, Curtin University, Perth, Australia; 3 School of Allied Health, Curtin University, Perth, Australia

**Keywords:** Machine learning, Abstract screening, Systematic reviews, Automation

## Abstract

**Background:**

Systematic reviews and meta-analyses provide the highest level of evidence to help inform policy and practice, yet their rigorous nature is associated with significant time and economic demands. The screening of titles and abstracts is the most time consuming part of the review process with analysts required review thousands of articles manually, taking on average 33 days. New technologies aimed at streamlining the screening process have provided initial promising findings, yet there are limitations with current approaches and barriers to the widespread use of these tools. In this paper, we introduce and report initial evidence on the utility of Research Screener, a semi-automated machine learning tool to facilitate abstract screening.

**Methods:**

Three sets of analyses (simulation, interactive and sensitivity) were conducted to provide evidence of the utility of the tool through both simulated and real-world examples.

**Results:**

Research Screener delivered a workload saving of between 60 and 96% across nine systematic reviews and two scoping reviews. Findings from the real-world interactive analysis demonstrated a time saving of 12.53 days compared to the manual screening, which equates to a financial saving of USD 2444. Conservatively, our results suggest that analysts who scan 50% of the total pool of articles identified via a systematic search are highly likely to have identified 100% of eligible papers.

**Conclusions:**

In light of these findings, Research Screener is able to reduce the burden for researchers wishing to conduct a comprehensive systematic review without reducing the scientific rigour for which they strive to achieve.

**Supplementary Information:**

The online version contains supplementary material available at 10.1186/s13643-021-01635-3.

Systematic reviews and meta-analyses are widely used to synthesise research findings across a body of literature to provide the highest level of evidence to inform policy and practice with regard to a specific research question [14, 30]. Well-executed systematic reviews conform to stringent guidelines (e.g. [25]) in an effort to produce transparent and methodologically rigorous syntheses of the available research [14]. As a research methodology, systematic reviews first appeared around the 1970s, and since that time their appeal has grown exponentially (e.g. by 2007 there were 11 systematic reviews published every day [4]). In 2017 alone, there were 11,000 reviews registered on PROSPERO, which represents an increase of 97.4% in just 6 years (from 285 in 2011 [32]). Suffice to say, systematic reviews are key to the methodological toolkit of the modern researcher.

The exponential increase in published research across the human sciences means that evidence syntheses now take longer and cost more money to conduct than they did when they first appeared over forty years ago [18]. Towards the end of the twentieth century, meta-analyses took on average around 1139 h to complete [1]. Recent estimates indicate that the time taken from registration of a systematic review to publication has more than doubled, with the total process from start to finish taking approximately 67.3 weeks [5]. The significant time demands required to produce a rigorous systematic review are associated with significant economic costs; recent estimates place this value at around USD 141,194 for a single paper [22]. Given the significant financial and time demands of systematic reviews and meta-analyses, advances in computational support are needed to optimise the review process without sacrificing reliability.

Researchers have focused on streamlining several of the steps within the systematic review process [14]. A review of automation technologies identified 14 tasks within the review process which have the potential to be automated (e.g. de-duplication and screening titles and abstracts [39]). These tools have been compiled in ‘the systematic review toolbox’ (http://systematicreviewtools.com/ [21]), an online catalogue of tools designed to facilitate the review process, which at the time of submission contains 189 different tools. The toolbox includes tools from multiple disciplines designed to support most, if not all, aspects of the review process. Although some of these tools are widely accepted and used (e.g. reference management software such as EndNote, RefWorks, and Zotero [20]), there is some scepticism on the part of reviewers to use more automated approaches to help with stages of a review that demand the greatest human resources (e.g. screening titles and abstracts [28]).

The task of screening titles and abstracts of articles is one of the most important aspects of the review process [34]. This stage of a systematic review involves reviewers screening the titles and abstracts of papers identified from the initial search — oftentimes in the tens of thousands [30] — to make an assessment on its relevance for inclusion, which typically takes around 30 s per paper [14]. Several tools have been developed to (semi)automate this screening process [16]. For example, a recent review of tools developed to support researchers through the review process identified 20 packages (of 22) that included help for the article screening stage [17]. Additionally, the screening stage was identified as the most frequently addressed of nine stages in the review process, potentially due to it being one of the most time intensive stages of the process (e.g. [29, 31, 40]). For example, a review of systematic reviews (*n* = 66) found that around 33 days (20%) of the average 164 person days taken to complete a review were spent on the screening process [15]. Despite the development of tools to facilitate the review process, their use has led to a negligible reduction on the time and money taken to produce a review [18]. Although not widely used at present, numerous researchers have advocated for a shift towards automation within the review process (e.g. [22, 27]).

Text mining and machine learning algorithms are an increasingly popular avenue of research to expedite the screening process [3]. Text mining is defined as ‘the process of discovering knowledge and structure from unstructured data (i.e. text)’ ([30]; p. 2). Machine learning utilises the mined text for training algorithms to create a classifier model based upon reviewers’ inclusion and exclusion decisions [40]. A review of studies using these approaches to expedite the screening process identified 44 papers, within which a number of computational methods where utilised (e.g. support vector machines, *k*-nearest neighbour, latent Dirichlet allocation, neural networks and active learning [30]). Machine learning approaches to the facilitation of title and abstract screening have been examined within many scientific disciplines, such as public health [24], animal studies [3] and genetics [42]. Thus, there is a thirst across academic disciplines for technological tools that can optimise the screening process for systematic reviews.

A key consideration for the acceptance of new technologies is evidence that they streamline the screening process efficiently and effectively. Rayyan, for example, is a free Web-based tool which employs a semi-automated approach using natural language processing methods (e.g. n-grams which involves representing text numerically by the occurrence of a word or sequence of words; *n* = 1 is referred to as unigrams, *n* = 2 is bigrams, *n* = 3 is trigrams) and support vector machines (i.e. a learning algorithm for developing classification and regression models) [31]. An independent examination of the tool found that, on average, 95% of the relevant articles were identified after screening 50% of all records, with the figure rising to 98% once 75% had been screened [29]. Abstrackr is another free Web-based tool that utilises active learning to periodically re-train the model as more data becomes available such as when a reviewer decides to include or exclude an article during their review; Abstrackr has been used in some capacity in over 50 reviews [42, 44]. Abstrackr extracts n-grams from article text and uses support vector machines to classify relevant or irrelevant articles [42, 44]. In two retrospective, independent evaluations of Abstrackr’s performance, the workload savings varied between 9 and 57% [36], and 9.5 and 88.4% [14]. Similar results have been observed with RobotAnalyst, a semi-automated Web-based tool which utilises active learning incorporating unigrams and support vector machines for classification [35]. A real-world evaluation of start to finish screening of 22 reference collections showed that RobotAnalyst saved between 7 and 71% of screening effort [35]. It should be noted that these results were based upon reviews within the public health domain, with a maximum size ranging between just 86 and 4964 references. The performance of three well-documented machine learning-based screening tools (Abstrackr, DistillerSR, and RobotAnalyst) was compared for both automated and semi-automated screening of titles and abstracts [13]. Although automation substantially reduced the time to complete screening (*M*_Total_ = 27.5 days), the risk of missing relevant studies was high (*M*_Total_ = 86.5%); results were improved for semi-automation with a lower risk of missing relevant studies (*M*_Total_ = 3%), whilst still saving a considerable amount of time (*M*_Total_ = 12.3 days).

ASReviewer is another free tool that employs active learning and a number of different machine learning and natural language processing methods. In a recent paper, the performance of ASReviewer was tested in a simulation study on four systematic reviews with labelled data [49]. Performance was assessed using work saved over random sampling (WSS [11]), which provides an indication of the time saved over manual screening in the form a percentage reduction in the number of papers needed to be screened. The level of WSS can be chosen dependent of the amount of recall required. For example, WSS@95 provides the work saved compared to traditional manual screening to find 95% of eligible papers. Due to the rigour associated with systematic reviews, researchers will want to find all relevant articles; thus, WSS was also set at WSS@100. Results demonstrated that with WSS@95 using ASReviewer saved screening between 67 and 92% of papers, with a mean saving of 83%. When recalling all relevant articles (WSS@100), ASReviewer saved screening between 38 and 93% of articles, with a mean saving of 61%. Rayyan and Abstrackr have also demonstrated considerable workload savings as measured by WSS. For example, when tested on a sample of 15 reviews with WSS@95, Rayyan produced an average workload saving of 49% ± 0.18 [31]. In a simulation study over two data sets, Abstrackr demonstrated a similar saving with WSS@100, whereby a workload saving of approaching 50% was found [43]. Together, the evidence supports semi-automated tools as viable and promising avenues to speed up the screening process, saving a considerable amount of time and money. Collectively, therefore, the available evidence provides promise for the use of machine learning for assisting screening processes, yet there remain additional gains to be made.

Despite promising findings from screening tools, the fast-paced nature of computing, software development and machine learning means that new methods and techniques may highlight previously unidentified limitations with tools that offer opportunities for innovations. First, existing programs have utilised traditional machine learning and natural language programming methods such as support vector machines and n-grams [30]. However, recent techniques have been shown to outperform these traditional methods and achieve state-of-the-art performance on many text mining tasks [12, 23, 41]. Second, some existing methods require reviewers to train the program by initially screening hundreds to thousands of articles, which limits the amount of time savings and therefore makes them unsuitable for use in smaller systematic reviews [30, 31]. Third, given the large variability in reliability estimates of existing semi-automated tools for the points at which they might offer the greatest time and cost savings, there is a need to develop reliable thresholds for when reviewers are able to stop screening [35]. Fourth, commercial software such as Covidence, whilst not offering machine learning-assisted abstract screening, provide additional features to better support the review process such as team or project management and conflict resolution for disagreements. Existing semi-automation tools evaluated in the literature are largely focused on abstract screening and do not provide these additional features ([31, 42, 44, 49]) which can limit their widespread adoption. Fifth, installation of the screening tool may be required on a computer or dedicated server ([30, 49]). Although this process is often simple enough for reviewers with computing expertise and familiarity with the technologies employed by the tool, it can present a barrier for adoption by non-expert users such as health researchers and students who likely represent the primary demographic who conduct systematic reviews. A cloud-hosted and web-based system such as Covidence reduces the barrier of entry for these types of users. Sixth, advancements in software development and open-source technologies has made it easier to develop visually appealing and intuitive user interfaces that can adapt to multiple platforms such as desktop, mobile and tablet devices (i.e. responsive design). Older generation screening tools developed only for a specific platform (e.g. Windows computer) would be uncompetitive with newer tools using modern technologies. Applying best practice software development techniques can also ensure positive user experiences particularly for larger and slower computational tasks such as training machine learning models for each systematic review.

These limitations and barriers to the widespread use of (semi)automation tools for title and abstract screening (e.g. trust, and ease of use) motivate the need for a tool that is reliable, effective and user-friendly. For such a tool to be considered seriously by researchers, clinicians and policy-makers, they require evidence to support the effectiveness of the semi-automation tool and the potential time savings associated with its adoption [28]. In this paper, we introduce Research Screener, a semi-automated tool to aid researchers with the screening of abstracts, addressing several of the limitations of existing tools highlighted previously. In so doing, we outline the development of Research Screener and present initial evidence on the utility of this tool within the review process.

## Methods

Research Screener (https://researchscreener.com) is a cloud-hosted Web application and algorithm that semi-automates abstract screening for systematic reviews. The algorithm applies deep learning and natural language processing methods to represent abstracts as text embeddings. Embeddings represent words or paragraphs within a document as a list of numbers rather than counting the occurrence of word sequences as with n-grams. These embeddings (i.e. list of numbers) are learnt for each collection of papers retrieved from a specific systematic review search that, when trained well, is able to represent the semantics and context of the abstract and its relation to other abstracts. Popular methods for training text embeddings include word2vec [23], Global Vectors for Word Representation-GloVe [33], paragraph embeddings [19] and attention and transformer models [12, 41]. Several natural language processing and machine learning methods were applied in developing the Research Screener algorithm such as n-grams, term frequency-inverse document frequency (tf-idf), word2vec, k-means clustering and support vector machines. The selected algorithm (best performing) applied paragraph embeddings [19], which was fine-tuned and validated against systematic reviews collected in this study. Note, the algorithm was selected based on a trade-off between performance (i.e. reducing the amount of abstracts that needed to be screen) and its computational complexity (i.e. how much computing resources/time is needed to run the algorithm). We chose a less complex method (i.e. paragraph embeddings vs. state-of-the-art transformer models) to reduce compute and storage cloud-hosting costs as well as to decrease the algorithm training time to ensure a better user experience for reviewers.

### Screening process

**Fig. 1:**
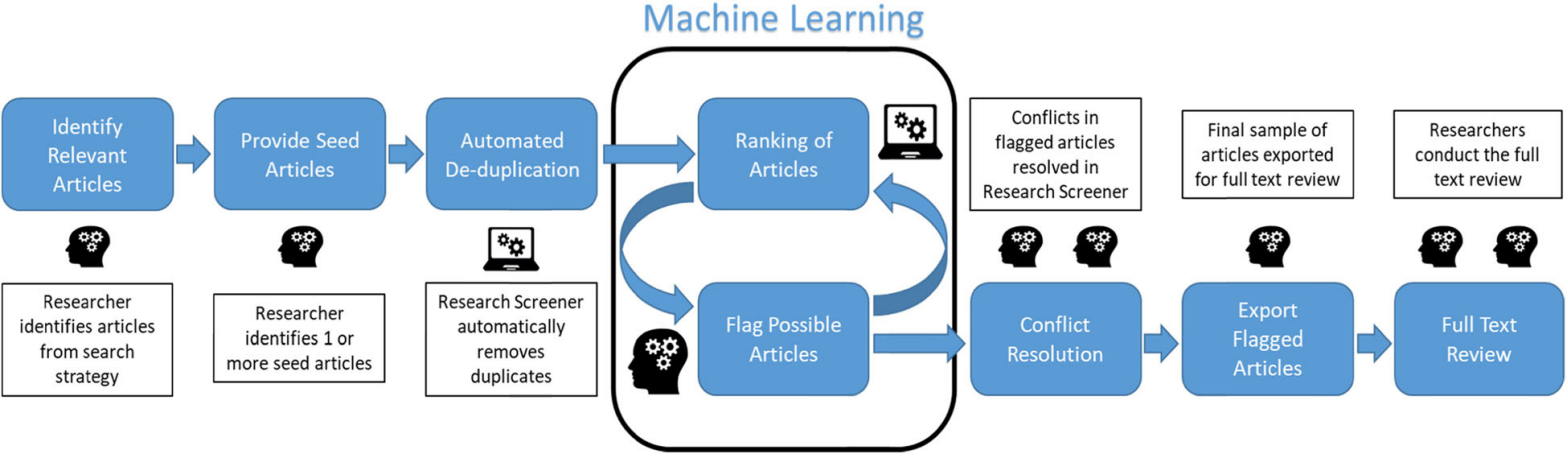
Research Screener assisted screening process. Head symbol = elements that require human input; computer symbol = elements augmented by Research Screener.

The algorithm is used to semi-automate the process of screening abstracts to identify relevant articles for a given systematic review. This process is described below and illustrated in Fig. 1:

1. Researchers provide two key pieces of required data for the screening process.
  - Total population of potentially eligible articles retrieved from their systematic review search strategy
  - At least 1 seed article assessed as highly relevant or representative of eligible studies based on the inclusion and exclusion criteria
2. Research Screener algorithm uses seed article(s) to rank articles by relevance
3. Loop
  - Research Screener presents the top 50 articles based on relevance (round 1)
  - Researchers screen the 50 articles (round 1) to determine if they are ir/relevant and therefore retained for full article screening. The researcher is given the option to flag each article if it is deemed as relevant.
  - Screening results are fed back into Research Screener to re-rank and determine the next top 50 most relevant articles (round 2, … *k*).
4. The screening process halts when either
  - All articles have been screened.
  - The research team reaches an evidence-based threshold where they can be certain all relevant articles have been identified. An evidence-based threshold of 50%, for example, would require that analysts with a total pool of 3,600 articles for scanning execute 36 rounds of title and abstract assessments in Research Screener.

The rationale for presenting articles in rounds of 50 is to provide the reviewer an opportunity to investigate the rankings generated by the current model before updating and re-ranking the remaining articles. This process is referred to as the exploration vs. exploitation trade-off problem commonly encountered in learning systems. Specifically, there are periods where you want to exploit the current state of the model to find relevant articles and other periods where you want to explore new rankings. In our preliminary experiments, we discovered that re-ranking too quickly (e.g. after reviewing 1, 5, 10, 20 articles) caused relevant articles to be re-ranked and discovered later resulting in inferior model performance. The threshold of 50 papers per round was chosen through experimentation on data from systematic reviews presented in this paper. An additional benefit of this approach is the reduced computation and improved user experience as the model ranks are only updated periodically rather than after each individual paper has been reviewed.

### Web application

**Fig. 2:**
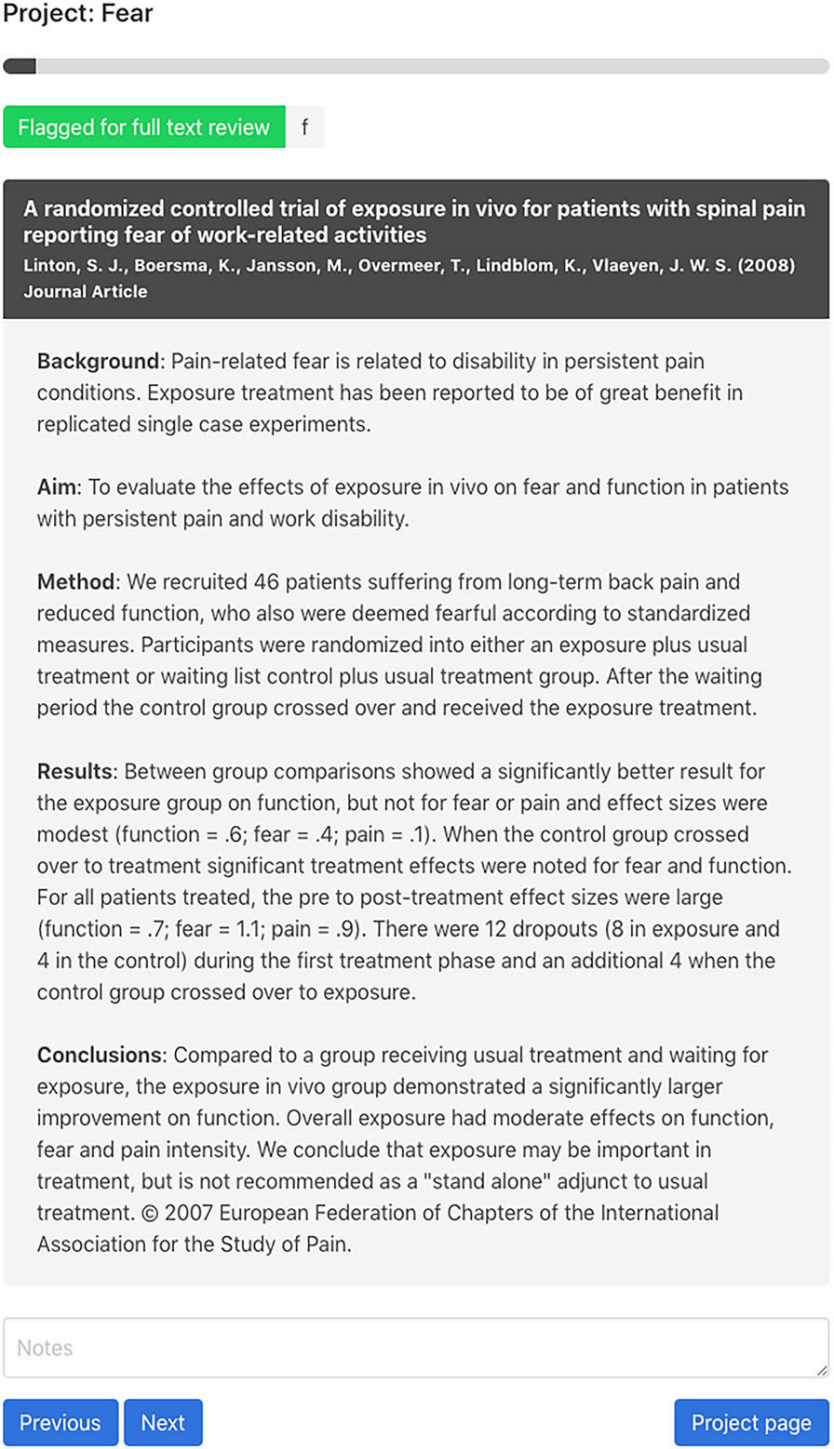
Research Screener web front end

Reviewers can access Research Screener via a Web browser (https://researchscreener.com). A screenshot of an example abstract screening webpage is shown in Fig. 2. Based on our review of existing screening tools, several useful features were also carried forward and developed in the Web application such as the following:

- Automated removal of duplicate articles.
- Ability for multiple researchers to collaborate on a review.
- Conflict resolution for disagreements.
- Exporting of de-duplication and screening results.
- Desktop, mobile and tablet device friendly user interface.

The process of conducting reviews with multiple researchers involves:

- Reviewers screening abstracts individually. Each reviewer is shown abstracts in a different order based on their decisions to include or exclude articles.
- The lead reviewer/project creator initiates the conflict resolution process when all or a sufficient number of articles have been screened by the team (e.g. rapid reviews may not require all articles to be screened).
- Conflicts (disagreements between reviewers) are identified and all team members are able to view, include or exclude and discuss each conflict using a commenting system.
- When the conflict resolution process is completed, the review team is able to export individual, team, conflicts and conflict resolution screening results.

### Validation

Three validation analyses were conducted on Research Screener’s algorithm to assess its real-world utility. In so doing, Research Screener’s algorithm has been fine-tuned and validated against several existing and completed systematic reviews (see Table 1). The papers column represents the total number of potentially relevant articles retrieved from a systematic review search strategy. The flagged column represents the number of papers flagged for full-text review in the abstract screening process. The final column refers to the number of articles in the published systematic review paper after full-text screening. For example, the sedentary systematic review started with a total of 932 papers, 22 of which were flagged for full-text review during abstract screening and only 8 were included in the final systematic review publication.

**Table 1:** Review papers used in validation analyses

Topic	Papers	Flagged	Final
Sedentary: overweight [45]	932	23	8
Low back pain: lifting [37]	2311	37	13
Low back pain: fear (under review)	2618	40	12
Lung cancer—preop [9]	395	7	5
Lung cancer—exercise [10]	891	31	10
Falls [8]	338	4	4
Acute pain (in press)	25,327	230	44
Team reflexivity [47]	13,376	140	20
Sexual health [46]	2415	69	18
Back pain education (under review)^a^	17,096	247	84
Ankyloglossia assessment (in development)^a^	1722	156	42

^a^Scoping review

### Analyses

#### Simulation analysis

An experiment was conducted to simulate researchers using the Research Screener algorithm for the nine systematic and two scoping reviews. In order to capture realistic simulation results, the set of flagged articles are used for each screening round to re-rank the next top 50 articles even though it is known from hindsight that some of the flagged articles do not make the final list after full-text review. The simulation is stopped when all of the final articles had been identified.

#### Interactive analysis

This test involved a researcher conducting the screening process for a review the traditional way followed by using Research Screener to complete the same process. The researcher had previous experience with conducting the screening stage of two systematic reviews in a traditional manner. The researcher reviewed all the articles keeping a detailed log, and then we evaluated the following:

- When all final papers were identified
- Time spent by the researcher using each method

#### Sensitivity analysis

We conducted a sensitivity analysis to provide insights on two open questions for using the algorithm:

1. *Threshold.* When should a researcher stop reviewing papers?
  - Can we be confident that the algorithm will find all eligible papers without screening all papers?
  - This question is analysed at 5% intervals; only screen 5%, 10%, 15%, 20% and 25% of papers.
  - Work saved over random sampling (WSS) was computed at both WSS@95 and WSS@100.
2. *Seed articles.* How important are the seed articles for producing good relevance rankings?
  - How many seed articles are needed?
  - Does the quality (combination) of seed articles matter?

## Results

### Simulation

The Research Screener algorithm was run against both systematic and scoping reviews (see Tables 2 and 3). We can see from the results that for systematic reviews the mean number of rounds of 50 articles required to find all eligible articles was 9.4, with rounds completed ranging from 1 to 36 (i.e. 50–1800 articles screened). This finding equates to a large saving in the number of papers that need to be screened using Research Screener; the mean percentage of papers that did not have to be reviewed was 79.3%, ranging between 68 and 96%. In terms of the two scoping reviews, the mean total of rounds completed was 87, ranging between 10 and 164. Total amount of papers not needed to be reviewed was similar for the two papers (60% and 62%).

**Table 2:** Papers screened to capture all relevant papers for systematic reviews

Topic	Papers^a^	Seeds^b^	Reviewed^c^	Not reviewed^d^
Sedentary: overweight	813	3 (8)	257 (6)	556 (68%)
Low back pain: lifting	2,249	5 (13)	283 (6)	1966 (87%)
Low back pain: fear	2,584	3 (12)	147 (3)	2437 (94%)
Lung cancer—preop	368	2 (5)	20 (1)	348 (95%)
Lung cancer—exercise	870	3 (10)	35 (1)	835 (96%)
Falls	306	1 (4)	12 (1)	294 (96%)
Acute pain	23,423	12 (44)	1275 (26)	22148 (95%)
Team reflexivity	13,376	2 (20)	1762 (36)	11614 (87%)
Sexual health	1686	3 (18)	239 (5)	1447 (86%)

^a^Total following removal of duplicates

^b^Number in brackets represents final sample of papers

^c^Number in brackets represents total rounds of 50 papers

^d^Number in brackets represents percentage of papers not reviewed

**Table 3:** Papers screened to capture all relevant papers for scoping reviews

Topic	Papers^a^	Seeds^b^	Reviewed^c^	Not reviewed^d^
Back pain education	16,506	5 (84)	6662 (164)	9844 (60%)
AG assessment	1230	5 (42)	470 (10)	760 (62%)

^a^Total following removal of duplicates

^b^Number in brackets represents final sample of papers

^c^Number in brackets represents total rounds of 50 papers

^d^Number in brackets represents percentage of papers not reviewed

### Interactive

To assess its real-world application, we compared Research Screener to a traditional manual approach for an assessment of the de-duplication and title and abstract screening stages of a review. One member of the research team (RL) who was conducting a systematic review and meta-analysis (Team Reflexivity) performed the traditional approach followed by Research Screener, keeping a detailed record of the time taken to complete each aspect of the review. The initial database search returned 18,102 articles. Following the importation of articles into Endnote, the first step undertaken was the removal of duplicates. Using a traditional manual approach [6], the de-duplication took approximately 7 h to complete, though with Research Screener this process was completed in a matter of seconds. The time taken on the de-duplication for manual screening is similar to findings reported in a review surveying 33 experienced reviewers (Mean_reviews completed_ = 4.30 ± 5.33), where they reported a mean total time of 1.37 days + 1.40 [15]. The manual process implemented here identified 5121 duplicates, whereas Research Screener identified and removed 4726 duplicates, equating to a discrepancy of 395. This discrepancy is due to the researchers manually performing de-duplication and removing non-exact matches (e.g. records that might contain extra whitespace, punctuation, a truncated author list), whereas Research Screener only removes exact matches to avoid potential errors in its automated de-duplication process. The records removed from the automated de-duplication process can be downloaded and cross-checked by the researchers. The researcher subsequently screened the titles and abstracts for potentially eligible articles (see Table 4). In total, the manual approach took 118 h over 19 days at an average of 6.21 h/day, equating to 15.73 FTE days. Over the 19 days the mean number of articles screened per day was 683.26, which equates to a mean of 825.13 per FTE day.

**Table 4:** Details of traditional title and abstract scanning

Day	Articles — start of day	Articles — end of day	Screened per day	h
1	12,981	12,747	234	3
2	12,747	12,013	734	7
3	12,013	11,220	793	7
4	11,220	10,216	1004	7
5	10,216	9191	1025	7
6	9191	8377	814	7
7	8377	7473	904	7
8	7473	6520	953	6
9	6520	5596	924	7
10	5596	4994	602	6
11	4994	4495	499	5
12	4495	3840	655	7
13	3840	3222	618	7
14	3222	2353	869	7
15	2353	1783	570	5
16	1783	1388	395	6
17	1388	793	595	6
18	792	292	500	7
19	292	0	292	4
Total	-	-	12981	118

Following completion of the traditional manual approach, RL used Research Screener to assess the total pool of potentially relevant articles (see Table 5). To recreate the traditional review only those papers that were taken forward for full-text review (149) in the traditional review were flagged by RL in Research Screener. RL stopped the screening process once all 20 papers identified via the manual search process had been found, which required examination of 1800 titles and abstracts only (i.e. 13.46% of the initial set of papers). With Research Screener, the screening process took 24 h over 4 days at an average of 6 h/day; 3.2 FTE days, with a mean of 450 articles screened per day; or 562.5 per FTE day. Thus, Research Screener saved 12.53 FTE days in screening time when compared with the traditional manual approach.

**Table 5:** Details of Research Screener title and abstract scanning

Day	Number screened per day	h
1	500	7
2	400	5
3	500	7
4	400	5
Total	1800	22

The savings in time associated with Research Screener translates into meaningful economic savings. The median postdoctoral researcher salary in the US is around USD 47,484, equating to just under USD 26/h [2]. Therefore, with regard to the comparison of manual screening versus Research Screener, the cost of the traditional screening would total around USD 3068, whereas using Research Screener would cost USD 624, providing a saving of USD 2444. Furthermore, Haddaway and Westgates’s (2019) review reported the mean number of titles and abstracts screenable per day to be 468.14 ± 128.22. If we apply this mean rate to the set of articles screened in the traditional approach from this study, it would equate to 27.73 FTE days spent screening. This would increase the costs of completing the screening stage to USD 5407.13, increasing savings to USD 4783.13.

### Sensitivity analyses

#### Threshold analysis

An important consideration when using a semi-automated method for conducting systematic reviews is whether or not researchers are required to read all the articles before they are confident that all relevant papers have been identified. This idea was explored in two sets of analyses. First, we examined how many of the final papers of systematic reviews were captured at 5% intervals of the total number of papers in the initial search in a sample of eight systematic reviews (see Table 6**)**. For example, in the low back pain-fear systematic review, all 12 final articles were identified after screening the first 5% of the total number of papers. However, for the sedentary-overweight systematic review, we see that 35% of the articles were needed to be screened before all final set of eight articles were identified. The percentage of articles needed to be screened to find all relevant articles ranged between 5 and 35% with a mean of 12.8%. Second, the WSS was computed at both WSS@95 and WSS@100 for each of the eight systematic reviews (see Table 6). At the 95% level, the average work saved over that of random sampling when using Research Screener was 89.1% ranging from 68 to 96%. This value equates to finding 95% of the eligible studies after screening between just  4 and 32% of the initial sample of papers. When looking at work saved over random sampling at the 100% level, the mean time saved using Research Screener was 88.7%, equating to finding all relevant papers after screening between only 4 and 32% of papers. These results suggest that with the use of Research Screener researchers can gain substantial savings in the time taken to complete the screening process.

**Table 6:** Percentage of papers screened to capture all relevant articles for systematic reviews

Topic	Papers	5%	10%	15%	20%	25%	30%	35%	WSS@95^a^	WSS@100^**b**^
Sedentary — overweight	813 (8)	6	7	7	7	7	7	8	68%	68%
Low back pain — lifting	2249 (13)	9	11	13	-	-	-	-	85%	85%
Low back pain — fear	2584 (12)	12	-	-	-	-	-	-	94%	94%
Lung cancer — preop	368 (5)	4	5	-	-	-	-	-	95%	95%
Lung cancer — exercise	870 (10)	10	-	-	-	-	-	-	96%	96%
Falls	306 (4)	4	-	-	-	-	-	-	96%	96%
Acute pain	23,423 (44)	43	44	-	-	-	-	-	96%	95%
Team reflexivity	13,376 (20)	17	19	20	-	-	-	-	90%	87%
Sexual health	1686 (18)	14	17	18	-	-	-	-	82%	82%

(Number) = final sample of papers

^a^Workload saved over random sampling at 95%

^b^Workload saved over random sampling at 100%

In terms of the threshold analysis for the two scoping reviews, a slightly different analysis was conducted. The number of rounds of 50 papers needed to be screened to find increments of 20% of the final sample of eligible papers was calculated (see Table 7). The workload saving over random sampling was again calculated at both WSS@95 and WSS@100. At the 95% level, Research Screener provided a mean work saved over random sampling of 70.5%, whereas at the 100% level a mean workload saving of 60% was obtained. These findings again emphasise the possible time savings researchers can benefit from when using Research Screener.

**Table 7:** Percentage of papers screened to capture all relevant papers for scoping reviews

Topic	Papers	20%^**a**^	40%	60%	80%	100%	WSS@95	WSS@100
Back pain education	16,506	2	5	9	18	134	76%	60%
AG assessment	1230	1	2	3	6	10	65%	60%

^a^Number of rounds of 50 articles screened to find the percentage of papers

#### Seed articles

A key feature of Research Screener is the requirement for seed articles—papers known to the research team that exemplify the key inclusion and exclusion criteria—to optimise the machine learning algorithm. This requirement begs the question regarding how many seed articles are optimal. We examined this question by varying the number and combination of seed articles used by Research Screener for the systematic reviews validated in this paper.

Results from the acute pain systematic review are shown in Table 8 (see supplementary material for results for other reviews). Seeds refer to the number of papers included for each experiment. The median, minimum and maximum numbers refer to the number of screening rounds (50 articles per round) required to discover the final set of articles included in the published systematic review. Finally, combinations refer to the number of possible combinations of seed articles. For example, there is only 1 combination when using all 12 seed articles, whereas there are 12 combinations for using 11 seed articles by leaving out a different seed article for each experiment run. As it is computationally expensive to run experiments on all combinations, we randomly sample and average the results over 12 runs for the acute pain systematic review.

**Table 8:** Sensitivity analysis of seed articles for acute pain systematic review

Seeds	Median	Minimum	Maximum	Combinations
12	26	26	26	1
11	26	26	26	12
10	26	26	26	66
9	26	26	26	220
8	26	26	26	495
7	26	26	26	792
6	26	26	26	924
5	26	26	26	792
4	26	26	26	495
3	26	26	26	220
2	26	26	26	66
1	26	18	27	12

When using only one seed article, we were able to find all the final papers on average in 26 rounds (26 × 50 = 1300 papers) which is the same round as using 2–12 seed articles. However, a comparison of the screening process of using one seed vs 12 seed articles shows that more relevant papers are ranked in earlier rounds but the one seed set quickly catches up (see Table 9). We obtained mixed results when analysing the other reviews (see supplementary materials). More specifically, the low back pain systematic review and the ankyloglossia assessment scoping review achieved poorer results when only provided one or two seed articles compared to the larger set whilst the other reviews were relatively insensitive to the number of seed articles provided.

**Table 9:** Comparison between the number of initial seed articles used

Round^a^	1 seed article			12 seed articles		
	Found^b^	Flagged^c^	Final^d^	Found^b^	Flagged^c^	Final^d^
0	0	1	1	0	12	12
1	13	14	5	23	35	22
2	24	38	13	25	60	25
3	25	63	19	18	78	26
4	11	74	21	14	92	27
5	19	93	21	12	104	27
6	15	108	24	11	115	28
7	13	121	26	13	128	30
8	9	130	28	8	136	32
9	6	136	28	8	144	34
10	9	145	30	6	150	34
11	4	149	30	4	154	35
12	4	153	30	7	161	36
13	1	163	32	4	165	36
14	4	167	34	3	168	36
15	10	177	35	3	171	36
16	1	178	36	4	175	36
17	0	178	36	4	179	36
18	2	180	36	2	181	36
19	3	183	36	1	182	36
20	3	186	36	2	184	36

^a^Rounds of 50 papers

^b^Number of papers flagged per round

^c^Cumulative number of papers flagged

^d^Cumulative number of final articles found

## Discussion

The screening process is one of the most important steps in conducting a comprehensive systematic review [34], yet is also the most time consuming element [15], with the research community calling for reliable tools to expedite the process (e.g. [18]; [48]; [26] [28];). In this paper, we introduced Research Screener and provided initial evidence regarding the utility of this tool in speeding up the screening process through simulation, interactive and sensitivity analyses. Our data support the viability of semi-automated tools to achieve workload savings over traditional screening methods, highlighting their utility in real-world environments. We demonstrated the benefits of Research Screener through simulation and real-world examples, revealing work load savings of between 60 and 96% across nine systematic reviews and two scoping reviews.

There is a growing interest in tools to facilitate and speed up the screening process (e.g. [3, 13, 21, 28]). A comparison of the simulation and sensitively analyses achieved from Research Screener with those of past work demonstrates a distinct advantage over existing tools with regard to WSS@95. For example, ASReviewer provides a mean saving of 83% over four reviews [49], Rayyan demonstrates a mean workload saving of 49% over 15 reviews [31], and RobotAnalyst provides a mean workload saving of 43% [35]. The sensitivity analysis with WSS@95 for Research Screener demonstrated a mean workload saving of 89%, providing an increased workload saving of between 6 and 46% over other screening tools. This advantage is also evident when looking at WSS@100, which can be seen as of greater importance due to the need for researchers to identify all possible articles to be included in a review. For example, in a simulation study using Abstrackr, a workload saving of around 45% was reported [43], whereas in a recent test of ASReviewer a mean saving of 61% was found [49]. Results generated using Research Screener provide a mean workload saving of 89%, again demonstrating a substantial saving over other methods (28% to 44%). Our findings provide initial evidence that the innovations embedded within Research Screener (e.g. enhanced algorithm) have translated into enhanced workload savings relative to existing tools to semi-automate the screening process.

The WSS provides a measure of the number of papers which do not need to be screened, yet equally important is the amount of time and money that can be saved from these efficiencies. Past work suggests that it takes between 30 [7] and 60 s [38] to screen each abstract. The most recent evidence obtained from a survey of 33 experienced reviewers [15] indicates that, on average, it takes 42.7 s to screen an abstract. Applying a time of 45 s per screened reference to the findings from the simulation and sensitivity analyses presented here would equate to a mean time saving of around 57.9 h (7.7 FTE days) over the nine systematic reviews (range 3.7 to 278.1 h). This saving is substantial relative the traditional method, with a mean saving of around 8 days of full-time work. These time savings translate into economic benefits. Using the mean postdoctoral salary of USD 26/h [2], the mean amount of money saved across the nine systematic reviews equates to USD 1508, ranging between USD 96 and 7232. A recent review including 66 systematic reviews reported the reviews to contain a mean of 8493 articles for the screening stage of the review [15]. Applying the 89% WSS@100 from the current paper to this mean obtained from 66 reviews equates to an average saving of 12.6 FTE days or USD 2457 when using Research Screener. These results are in line with the findings from the interactive analysis, which also saw a substantial time (12.5 FTE days) and financial (USD 2444) saving over the traditional screening approach. Taken together, these results demonstrate the significant time and financial savings that can be achieved via Research Screener to semi-automate the screening process.

One of the main challenges with the application of existing tools is the development of a reliable threshold of when to stop the screening process [35]. As researchers want to identify all relevant papers to ensure the review is as rigorous as possible, results of the WSS@100 can help to identify a reliable point at which researchers can stop screening with the assurance that 100% of relevant articles have been identified. Results from our sensitivity analyses (threshold analysis) demonstrate that, across the eleven reviews, all relevant papers were identified after screening between 4 and 40% of articles. Therefore, even by setting a conservative level of screening 50% of articles, systematic reviewers can achieve substantial time and financial savings using Research Screener.

### Strengths and limitations

The workload associated with the screening process in producing systematic reviews is a widely known problem. Although results across all reviews were supportive of the effectiveness of Research Screener, the sample of nine systematic reviews and two scoping reviews may be considered small. Furthermore, the majority of the reviews fall within the health sciences domain (10 of 11), meaning these time and cost savings may not generalise well to other disciplines. Nevertheless, the reviews included as part of the empirical analyses ranged from 306 to 23,423 initial papers, with substantial workload savings seen across reviews regardless of size. Within the interactive analysis, RL conducted both the traditional and manual screening flagging only those articles that were taken to full-text review in the traditional screening process. This approach could have affected the prioritisation of papers due to focus on only the articles taken to full texts, making it prudent to conduct further interaction analyses using a separate researcher for each aspect. Furthermore, using an independent reviewer to conduct the screening process may be necessary to counteract any learning effect artefacts. Another limitation with the machine learning prioritisation algorithm design used by Research Screener may be that researchers may become complacent when screening articles later in the process. As relevant articles are front ended, researchers may naturally perceive articles that are presented later in the process as less relevant. The researcher must stay vigilant throughout the process as eligible articles may appear later in the process which would be surrounded by those that are ineligible. However, the functionality of Research Screener to be able to compare results between researchers would help to mitigate the chances of relevant articles being missed.

## Conclusions

Due to the ever-growing body of academic research, the task requirements of conducting systematic reviews is likely to increase substantially over time [4]. Based upon the findings of this initial validation of a semi-automated machine learning-powered screening tool, the results provide initial confidence in the potential real-world workload savings that can be achieved through the use of Research Screener. Research Screener can help to reduce the burden for researchers wishing to conduct a comprehensive systematic review without reducing the scientific rigour for which they strive to achieve. We hope these findings may help the integration of machine learning tools into the review process enabling a more wide spread use and acceptance of these tools.

## Supplementary Information

**Additional file 1: Table S1**. Sensitivity analysis of seed articles for Sedentary systematic review. **Table S2**. Sensitivity analysis of seed articles for Low back pain: lifting review. **Table S3**. Sensitivity analysis of seed articles for Low back pain: fear review. **Table S4**. Sensitivity analysis of seed articles for Lung cancer - preop review. **Table S5**. Sensitivity analysis of seed articles for Lung cancer - exercise review. **Table S6**. Sensitivity analysis of seed articles for Falls review. **Table S7**. Sensitivity analysis of seed articles for Team reflexivity review. **Table S8**. Sensitivity analysis of seed articles for Sexual Health review. **Table S9**. Sensitivity analysis of seed articles for Back pain education review. **Table S10**. Sensitivity analysis of seed articles for AG Assessment review.

## Data Availability

The raw data used in this study are the abstracts from the initial searches in the examined systematic reviews and are subject to copyright from their associated journals.

## Abbreviations

FTE: Full-time equivalent
USD: US Dollar
WSS: Work saved over random sampling
WSS@95: Work saved over random sampling to find 95% of eligible papers
WSS@100: Work saved over random sampling to find 100% of eligible papers
