# Supplementary material for: Research Screener: a machine learning tool to semi-automate abstract screening for systematic reviews
Source: Syst Rev. 2021 Apr 1;10:93. doi: 10.1186/s13643-021-01635-3 (PMC8017894; doi:10.1186/s13643-021-01635-3)
Supplement: Supplementary file 1 — Additional file 1: Table S1. Sensitivity analysis of seed articles for Sedentary systematic review. Table S2. Sensitivity analysis of seed articles for Low back pain: lifting review. Table S3. Sensitivity analysis of seed articles for Low back pain: fear review. Table S4. Sensitivity analysis of seed articles for Lung cancer - preop review. Table S5. Sensitivity analysis of seed articles for Lung cancer - exercise review. Table S6. Sensitivity analysis of seed articles for Falls review. Table S7. Sensitivity analysis of seed articles for Team reflexivity review. Table S8. Sensitivity analysis of seed articles for Sexual Health review. Table S9. Sensitivity analysis of seed articles for Back pain education review. Table S10. Sensitivity analysis of seed articles for AG Assessment review. [file 13643_2021_1635_MOESM1_ESM.docx]

Table S1. *Sensitivity analysis of seed articles for Sedentary systematic review.*

| **Seeds** | **Median*** | **Minimum*** | **Maximum*** | **Combinations** |
| --- | --- | --- | --- | --- |
| 3 | 6 | 6 | 6 | 1 |
| 2 | 6 | 6 | 6 | 3 |
| 1 | 6 | 6 | 7 | 3 |

*Note*: * = Rounds of 50 papers to find all papers

Table S2. *Sensitivity analysis of seed articles for Low back pain: lifting review.*

| **Seeds** | **Median*** | **Minimum*** | **Maximum*** | **Combinations** |
| --- | --- | --- | --- | --- |
| 5 | 6 | 6 | 6 | 1 |
| 4 | 8 | 7 | 22 | 5 |
| 3 | 8 | 7 | 22 | 10 |
| 2 | 22 | 9 | 22 | 10 |
| 1 | 22 | 9 | 22 | 5 |

*Note*: * = Rounds of 50 papers to find all papers

Table S3. *Sensitivity analysis of seed articles for Low back pain: fear review.*

| **Seeds** | **Median*** | **Minimum*** | **Maximum*** | **Combinations** |
| --- | --- | --- | --- | --- |
| 3 | 3 | 3 | 3 | 1 |
| 2 | 7 | 3 | 9 | 3 |
| 1 | 9 | 7 | 9 | 3 |

*Note*: * = Rounds of 50 papers to find all papers

Table S4. *Sensitivity analysis of seed articles for Lung cancer - preop review.*

| **Seeds** | **Median*** | **Minimum*** | **Maximum*** | **Combinations** |
| --- | --- | --- | --- | --- |
| 2 | 1 | 1 | 1 | 1 |
| 1 | 1 | 1 | 2 | 2 |

*Note*: * = Rounds of 50 papers to find all papers

Table S5. *Sensitivity analysis of seed articles for Lung cancer - exercise review.*

| **Seeds** | **Median*** | **Minimum*** | **Maximum*** | **Combinations** |
| --- | --- | --- | --- | --- |
| 3 | 1 | 1 | 1 | 1 |
| 2 | 3 | 1 | 3 | 3 |
| 1 | 3 | 2 | 3 | 3 |

*Note*: * = Rounds of 50 papers to find all papers

Table S6. *Sensitivity analysis of seed articles for Falls review.*

| **Seeds** | **Median*** | **Minimum*** | **Maximum*** | **Combinations** |
| --- | --- | --- | --- | --- |
| 1 | 1 | 1 | 1 | 3 |

Table S7. *Sensitivity analysis of seed articles for Team reflexivity review.*

| **Seeds** | **Median*** | **Minimum*** | **Maximum*** | **Combinations** |
| --- | --- | --- | --- | --- |
| 2 | 36 | 36 | 36 | 1 |
| 1 | 36 | 36 | 36 | 2 |

*Note*: * = Rounds of 50 papers to find all papers

Table S8. *Sensitivity analysis of seed articles for Sexual Health review.*

| **Seeds** | **Median*** | **Minimum*** | **Maximum*** | **Combinations** |
| --- | --- | --- | --- | --- |
| 3 | 5 | 5 | 5 | 1 |
| 2 | 6 | 5 | 6 | 3 |
| 1 | 6 | 5 | 6 | 3 |

*Note*: * = Rounds of 50 papers to find all papers

Table S9. *Sensitivity analysis of seed articles for Back pain education review.*

| **Seeds** | **Median*** | **Minimum*** | **Maximum*** | **Combinations** |
| --- | --- | --- | --- | --- |
| 5 | 134 | 134 | 134 | 1 |
| 4 | 135 | 133 | 135 | 5 |
| 3 | 134 | 134 | 136 | 10 |
| 2 | 135 | 134 | 135 | 10 |
| 1 | 135 | 134 | 136 | 5 |

*Note*: * = Rounds of 50 papers to find all papers

Table S10. *Sensitivity analysis of seed articles for AG Assessment review.*

| **Seeds** | **Median*** | **Minimum*** | **Maximum*** | **Combinations** |
| --- | --- | --- | --- | --- |
| 5 | 10 | 10 | 10 | 1 |
| 4 | 25 | 11 | 25 | 5 |
| 3 | 25 | 11 | 25 | 10 |
| 2 | 25 | 25 | 25 | 10 |
| 1 | 25 | 25 | 25 | 5 |

*Note*: * = Rounds of 50 papers to find all papers
